# Tako-Tsubo Syndrome Triggered by a Fibroscopy: Case Report

**DOI:** 10.7759/cureus.52420

**Published:** 2024-01-17

**Authors:** Jaouad Nguadi, Raid Faraj, Zaynab Mouhib, Zouhair Lakhal, Hicham Bouzelmat

**Affiliations:** 1 Cardiology, Mohammed V Military Hospital, Mohamed V University, Rabat, MAR; 2 Cardiology, Ibn Sina Hospital University, Mohammed V University, Rabat, MAR

**Keywords:** clinical case report, transthoracic echocardiogram, systolic heart failure, tako-tsubo cardiomyopathy (ttc), atypical chest pain, ventriculography

## Abstract

Tako-Tsubo cardiomyopathy, also called stress cardiopathy, is a rare syndrome characterized by transient regional systolic dysfunction. It can mimic myocardial infarction but the absence of coronary obstruction allows to redress the diagnosis. Its pathogenesis is not well understood. However, the role of physical or emotional stress has often been associated with this pathology.

Here we report, a rare case of a 63-year-old female, with no cardiac risk factors, who presented Tako-Tsubo syndrome after a fibroscopy. This case aims to show that Tako-Tsubo syndrome should be suspected in patients, especially women, with no cardiac risk factors, who present acute chest pain in the context of physical or emotional stress, after excluding differential diagnoses.

## Introduction

Tako-Tsubo cardiomyopathy (TTC) is defined as a transient left ventricle (LV) dysfunction triggered by a stressful event [1]. It was first identified in Japan in the 1990s and was named after Japanese octopus traps (takotsubo) that are shaped similarly to the heart of affected individuals in its typical form [2]. TTC is also called “stress cardiomyopathy” or “apical ballooning syndrome” and mostly affects elderly women [3]. It classically involves apical ballooning due to apical akinesis or hypokinesis with preserved or hypercontractile basal segments, but there are other types of LV involvement that are rare: inverted, midventricular, or basal TTC. The genesis of TTC is complicated and is subject to numerous hypotheses, some of which are still being investigated [3-4]. One of the hypotheses that has long been debated is the cardiovascular response to a sudden surge of circulating catecholamines. However, emotional, physiological, and even environmental stressors are thought to be involved in its genesis. American Heart Association described TTC as a secondary cardiomyopathy [5-6]. Transthoracic echocardiography (TTE) is the method of choice for the evaluation of LV function and visualization of symmetric regional wall motion abnormalities (WMA) [7]. Takotsubo patients, usually, have a good improvement in LV function, making it a benign condition most of the time [6]. Although the prognosis is favorable, these patients require treatment and close monitoring in the acute phase to prevent fatal complications. Tako-tsubo syndrome should be known by all practitioners and considered as a differential diagnosis in patients presenting with acute coronary syndrome (ACS), especially after physical or emotional stress.

## Case presentation

**Figure 1:**
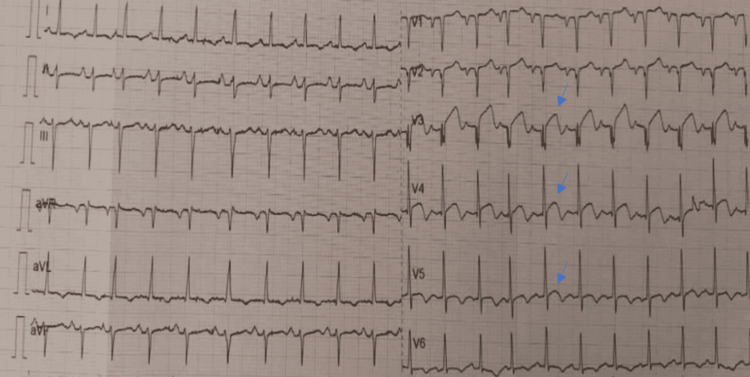
EKG findings ST-segment elevation in anterior leads

We present the case of a 63-year-old female patient, with no cardiac risk factors. The patient was admitted for gastritis, the clinical findings were unremarkable, and the laboratory workup showed anemia at 11 g/dl. Upon entering the endoscopy room, the patient developed acute chest pain. An electrocardiogram was performed and revealed ST-segment elevation in anterior leads (Figure 1), leading to the diagnosis of myocardial infarction.

**Figure 2:**
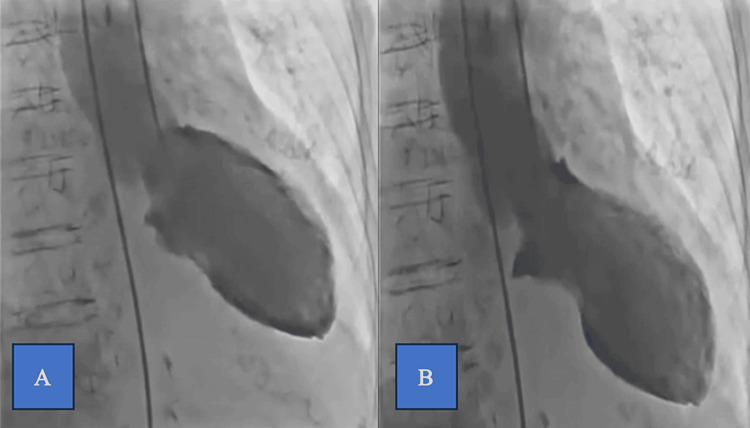
Ventriculography findings (RAO view) A: end diastole, B: end systole

The TTE showed apical ballooning with akinesia of the apex, basal hyperkinesis, and an impaired function of the LV with an estimated ejection fraction of 34%. Non-obstructive coronary artery disease and basal hyperkinesis with apical akinesis of the LV were detected by coronary angiography with ventriculography (Figure 2). High-sensitivity cardiac troponin (hs-cTn) values were 40 ng/l (normal values are below 14 ng/l).

The management included an angiotensin-converting enzyme inhibitor (ramipril 2.5 mg per day) and beta-blocker (bisoprolol 5 mg per day) therapy. A 3-month TTE check-out showed a complete recovery with normal wall motion and systolic LV function at 55%.

## Discussion

In recent years, a clinical entity known as TTC (transient apical ballooning syndrome of the LV) has been identified in Japan and more recently in Caucasian subjects. TTC is characterized by significant and transient reversible LV dysfunction without clear explanations from coronary ischemia, aortic valve disorders, or myocarditis [3]. Typically triggered by physical or emotional stress, TTC predominantly affects postmenopausal women, mimicking ACS with chest pain, electrocardiographic abnormalities, and a moderate increase in cardiac enzymes. Coronary angiography reveals no stenosis, presenting a “takotsubo” appearance on ventriculography.

While left ventricular function is usually impaired, it often normalizes within days or weeks (< 21 days) due to LV WMA [8-9]. Echocardiograms, crucial for TTC diagnosis, show symmetrical regional abnormalities extending beyond coronary artery territories (midventricular segments of the anterior, inferior, and lateral walls) [6,10]. TTC, initially described as apical ballooning, involves hyperkinetic left ventricular base, midventricular hypokinesis, and apical akinesis or dyskinesis, causing apical distention and impaired systolic function.

Estimating TTC frequency has been challenging, with recent assessments indicating 1-2% of suspected acute myocardial infarction cases [11]. Pathophysiological mechanisms include the vascular spasm hypothesis, microvascular thrombosis, and abnormally high plasma concentrations of catecholamines. As part of ACS, TTC affects a specific population prone to bleeding complications during thrombolytic therapy. The value of fibrinolysis in TTC patients remains unevaluated, though some have received fibrinolytic agents, emphasizing the importance of prompt coronary angiography in the benefit/risk assessment.

In 2018, the European Society of Cardiology introduced an algorithm for TTC diagnosis and management using the inter-TAK diagnostic score. For patients with ST-segment elevation, urgent coronary angiography is recommended. In the absence of ST-segment elevation and suspicion of TTC, the inter-TAK score guides further evaluation. Patients with low or intermediate probability (≤70 points) should undergo coronary angiography, while those with high probability (>70 points) may opt for TTE as a first-line procedure. In stable patients with typical WMA, coronary computed tomography angiography may rule out coronary disease. Our case involves a unique presentation of TTC during endoscopy, highlighting the importance of preparing patients for invasive exams. The prognosis for TTC is generally favorable without treatment, but short-term complications, including heart failure, cardiogenic shock, and ventricular arrhythmias, may occur. The long-term prognosis remains uncertain despite LV function normalization due to limited hindsight [12-13].

## Conclusions

In the past years, TTC has become a well-known type of acquired cardiomyopathy, mostly following stressful situations, and occurring especially in elderly women. The pathophysiology remains unknown, although there are some hypotheses, most of which are currently being explored. Clinicians should have awareness of this syndrome and its usual clinical manifestations. TTC should be considered in the differential diagnosis of ACS in patients presenting with chest pain or dyspnea.
